# Safety and Efficacy of Aspirin and Indobufen in the Treatment of Atherosclerotic Diseases: Systematic Review and Meta-Analysis

**DOI:** 10.2196/75363

**Published:** 2025-08-20

**Authors:** Wenhao Pan, Linger Guan, Haicheng Zhang

**Affiliations:** 1 Department of Cardiology, Peking University People's Hospital, 11 Xizhimen S St, Xicheng District, Beijing, 100044, China, 86 13366157815

**Keywords:** atherosclerotic disease, antiplatelet therapy, aspirin, indobufen, PRISMA

## Abstract

**Background:**

The pathogenesis of atherosclerotic thrombosis primarily involves platelet activation and aggregation, making antiplatelet therapy the cornerstone of treatment for such diseases.

**Objective:**

This meta-analysis aimed to compare the safety and efficacy of aspirin and indobufen in antiplatelet therapy for patients with atherosclerotic diseases.

**Methods:**

We searched the Cochrane Library, PubMed, Embase, Web of Science, and the Chinese Wanfang databases. The literature was screened according to predefined inclusion and exclusion criteria. Risk ratio (RR) was used to assess the magnitude of risk associated with exposure and our inclusion criteria are as follows: (1) the study population comprised adults (aged 18 years and older) with coronary heart disease caused by coronary artery atherosclerosis or stroke caused by intracranial atherosclerosis; (2) the intervention was represented by indobufen in the study groups versus aspirin in the control groups; (3) the primary outcome was the incidence of major adverse cardiovascular and cerebrovascular events or any bleeding or Bleeding Academic Research Consortium 2/3/5 bleeding; (4) the secondary outcomes were cardiovascular death, myocardial infarction, and ischemic stroke; adverse cardiovascular events such as coronary thrombus reformation, heart failure, myocardial infarction, stroke, angina pectoris, and cardiovascular death; stroke; myocardial infarction; and cardiovascular death; and (5) the studies were randomized clinical trials with either crossover or parallel designs or prospective observational trials.

**Results:**

Eighteen trials with a total of 12,981 patients were included in this study. Compared to aspirin, indobufen reduced the risk of (1) bleeding events (RR 0.54, 95% CI 0.41‐0.71; *P*<.0001), (2) Bleeding Academic Research Consortium 2/3/5 bleeding (RR 0.50, 95% CI 0.26‐0.94; *P*=.03), (3) adverse cardiovascular events (RR 0.43, 95% CI 0.30‐0.61; *P*<.00001), and (4) myocardial infarction (RR 0.60, 95% CI 0.41‐0.89; *P*=.01). However, there were no significant differences between the 2 groups in terms of major adverse cardiovascular and cerebrovascular events, stroke, or cardiovascular mortality.

**Conclusions:**

Compared with aspirin, indobufen demonstrated better safety and was not inferior to aspirin in terms of efficacy, with superior results in some aspects (eg, fewer risks of adverse cardiovascular events and myocardial infarction). Further studies with larger sample sizes or longer follow-up periods may provide additional evidence.

## Introduction

Atherosclerotic diseases include a variety of conditions caused by the buildup of plaque within arteries, leading to their narrowing and hardening [1]. This type of plaque is primarily composed of cholesterol, lipids, and other substances, which progressively restrict blood flow to key organs. Common manifestations include coronary atherosclerosis, intracranial atherosclerosis, and carotid artery and peripheral artery atherosclerotic disease. They increase the risk of cardiovascular events such as heart attacks and strokes. Major risk factors include elevated cholesterol, hypertension, diabetes, smoking, and obesity [2].

The pathogenesis of atherothrombosis is mainly the activation and aggregation of platelets, and platelet reactivity might be associated with the severity of atherosclerosis [3]. Therefore, antiplatelet therapy is the cornerstone of treatment. This activation triggers the release of various signaling molecules, including thromboxane A2 (TXA2), which is synthesized from arachidonic acid via the cyclooxygenase-1 (COX-1) enzyme [4]. TXA2 plays a critical role in amplifying the platelet activation process by promoting the recruitment of additional platelets to the injury site, thereby increasing platelet aggregation [5]. The importance of the COX-1 enzyme in platelet activation is evident in the mechanism of antiplatelet drugs such as aspirin and indobufen [6,7]. This disruption in the TXA2 pathway significantly lowers the risk of thrombus formation, making COX-1 inhibitors essential for the prevention and treatment of atherosclerotic disease. In summary, the COX-1/TXA2 pathway is central to platelet aggregation, and its inhibition is a key therapeutic strategy for managing atherosclerotic diseases [8].

In patients with atherosclerotic disease, the primary benefit of antiplatelet therapy is the reduction in adverse cardiovascular events related to arterial stenosis, ischemia, and occlusion [9]. The main adverse reaction, however, is mucosal bleeding caused by platelet aggregation inhibition [10]. Given that aspirin has a greater risk of intracranial and gastrointestinal bleeding than other antiplatelet agents do and that some patients may develop resistance, recent studies have focused on alternatives to aspirin [11,12]. Indobufen is gradually attracting the attention of clinicians because of the potential safety profile. Currently, Chinese guidelines acknowledge the feasibility of indobufen as an antiplatelet agent but do not consider it a replacement for aspirin [13]. In contrast, indobufen is less frequently mentioned as a treatment option for patients in the European and American guidelines due to the insufficient clinical evidence. Its further use on a global scale may require additional clinical evidence to attract more attention from clinicians.

In the evaluation of the efficacy and safety of antiplatelet therapy for patients with atherosclerotic disease, commonly assessed outcomes include the incidence of major adverse cardiovascular and cerebrovascular events (MACCEs) [14,15]. MACCEs is a composite end point comprising cardiovascular death, nonfatal myocardial infarction (MI), clinically driven repeated revascularization, definite or probable stent thrombosis, and nonfatal ischemic stroke. Moreover, safety can be evaluated on the basis of the incidence of bleeding events. The Bleeding Academic Research Consortium (BARC) defines the primary safety end point as type 2, 3, or 5 bleeding, also known as major bleeding [16]. Another safety outcome is any bleeding, including mild to severe bleeding.

This paper comprehensively reviewed clinical trials and cohort studies on the efficacy and safety of indobufen in the treatment of atherosclerotic diseases such as stroke and coronary heart disease (CHD), with aspirin as the control. Through meta-analysis, we aimed to integrate existing clinical studies to explore the incidence of MACCEs during the treatment of patients with atherosclerosis with indobufen, evaluate its efficacy, and assess the safety of the drug through the incidence of mucosal bleeding to provide a reference for the clinical application of indobufen and emphasize the necessity of further large-sample prospective studies. Our study aims to contribute to this evidence of indobufen, and we hope that our work will further inform the clinical safety and efficacy of indobufen, inspiring more global clinical research to validate these findings and ultimately contribute to the optimization of pharmacological treatment for atherosclerotic disease.

## Methods

### Literature Review Design and Literature Search

This study is registered with PROSPERO (CRD42024588250) and was conducted in accordance with the 2020 version of the PRISMA (Preferred Reporting Items for Systematic Reviews and Meta-Analyses) statement (the PRISMA checklist is provided in Checklist 1) [17]. Four English databases (Cochrane Library, PubMed, Embase, and Web of Science) and 1 Chinese database (Wanfang) were searched, and the publication deadline for the literature was October 1, 2024. We searched each database to identify MeSH (Medical Subject Headings) (eg, “aspirin,” “indobufen,” and “clinical trial”) and related free terms (eg, Acetylsalicylic Acid*, Acetysal*, Acylpyrin*, Aloxiprimum*, Colfarit*, Ibustrin*, controlled trial*, and Trial*). The search strategy was developed via a combination of MeSH terms and related free terms. Moreover, the references of all included studies were browsed and screened to complement the eligible literature not retrieved. Owing to the limited number of randomized controlled trials (RCTs) in the relevant field, we also included eligible well-conducted cohort studies in our analysis to enhance statistical power and generalizability. This approach of combining RCTs and nonrandomized studies is acceptable when the objective is to evaluate both efficacy and effectiveness in broader clinical settings [18].

### Study Selection

For inclusion in the systematic review and meta-analysis, we selected clinical trials that investigated the antiplatelet impact of indobufen and aspirin and their safety in human participants. The following inclusion criteria were applied: (1) the study population comprised adults (aged 18 years and older) with CHD caused by coronary artery atherosclerosis or stroke caused by intracranial atherosclerosis; (2) the intervention was represented by indobufen in the study groups versus aspirin in the control groups; (3) the primary outcome was the incidence of MACCEs or any bleeding or BARC 2/3/5 bleeding; (4) the secondary outcomes were cardiovascular death, MI, and ischemic stroke; adverse cardiovascular events such as coronary thrombus reformation, heart failure, MI, stroke, angina pectoris, and cardiovascular death; stroke; MI; and cardiovascular death; and (5) the studies were randomized clinical trials with either crossover or parallel designs or prospective observational trials. We have supplemented the secondary outcome measures from the registered protocol in order to enhance the guiding value of clinical practice. Our exclusion criteria were as follows: (1) lack of control group studies, (2) no studies reporting cardiovascular or cerebrovascular events or bleeding during the follow-up or research group period, (3) follow-up time of less than 6 months or no mention of follow-up time, and (4) case control studies, cross-sectional studies, ecological studies, case reports, and other descriptive studies. Reviews, conference abstracts, and commentaries were also excluded. Two authors (WHP and LEG) independently searched the appropriate studies from all the databases. Two authors (WHP and LEG) independently screened the studies and removed the papers that were duplicates or not relevant after carefully screening the titles and abstracts. In the next step, we further independently examined the remaining trials and excluded the ineligible trials according to the full text. We resolved disagreements between the authors by discussion. And a third researcher (HCZ) further resolved any conflicts.

### Data Extraction

Two researchers (WHP and LEG) independently extracted the following characteristics from the trials via a standardized data extraction form: year of publication, lead author, country of publication, total sample size of the trial in the intervention and control groups, duration of follow-up (months), age at entry, participants’ sex percentage, drug dosage (mg per day), and sample size for the occurrence of outcome indicators.

### Statistical Analysis

Our data were managed via Excel, and we used Review Manager 5.3 (The Cochrane Collaboration) and Stata SE 18.0 (Stata Corp LLC) to combine and analyze outcomes from the selected trials. All the data were pooled to calculate the risk ratio (RR) and 95% CIs via a fixed-effects model. Between-trial heterogeneity was assessed by using an *I*^2^ test, and a value of >50% was regarded as considerable heterogeneity. If heterogeneity was high, we used a random-effects model. Statistical significance was defined as a *P* value <.05. If the number of pooled studies was greater than 10, publication bias was surveyed by visual inspection of the funnel plot and Egger test.

### Assessment of Risk of Bias and Sensitivity Analysis

We divided all the included studies into RCTs and non-RCT trials. Two researchers (WHP and LEG) independently assessed the quality of RCTs on the basis of the Cochrane risk-of-bias criteria according to the following criteria [18]: (1) allocation concealment, (2) random sequence generation, (3) blinding of participants and personnel, (4) blinding of outcome assessment, (5) incomplete outcome data, (6) selective reporting, and (7) other probable sources of bias. The Newcastle‒Ottawa Scale was used to evaluate risk of bias for non-RCTs across domains such as selection, intervention classification and comparability, missing data, outcome measurement, and result reporting [19]. Studies with a score of ≥7 were considered high-quality studies. We performed leave-one-out sensitivity analyses to test the robustness of the results.

## Results

### Results of the Literature Search

**Figure 1.**
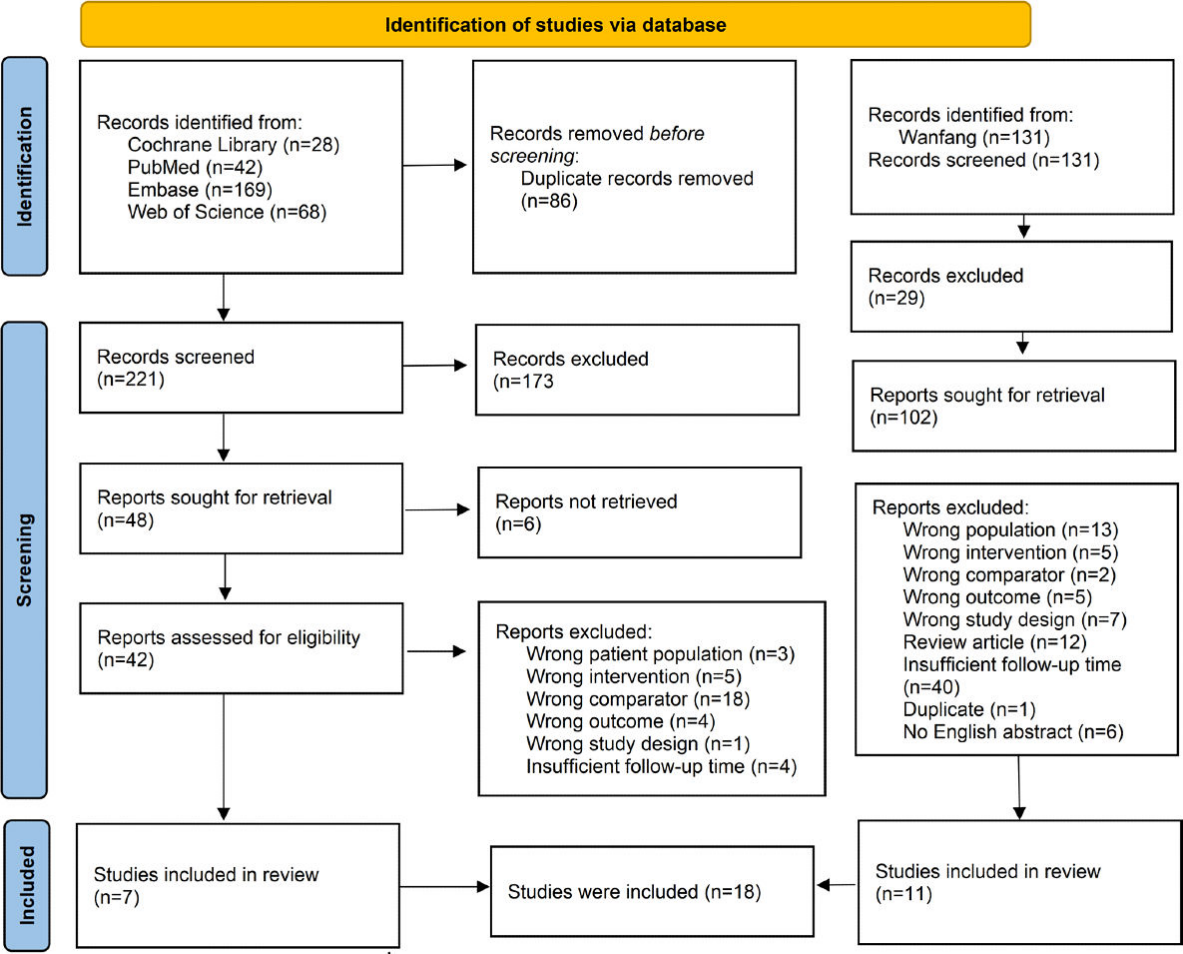
PRISMA (Preferred Reporting Items for Systematic Reviews and Meta-Analyses) flow diagram for the search and selection of included studies.

As reported in Figure 1, 307 papers from the English database were initially retrieved. A total of 86 duplicate studies were removed. Among the remaining 221 papers, 173 were excluded after the titles and abstracts were carefully screened. In the next step, 48 trials were further examined, and 41 additional trials were excluded for the following reasons: the population studied in 3 papers did not meet the inclusion criteria, the intervention methods used in 5 papers did not meet the requirements, 4 studies did not report the required outcome measures, 18 studies did not provide a proper comparison with aspirin, 1 study’s design method did not meet the criteria, 4 papers had a follow-up time of less than 6 months or did not mention the follow-up time, and 6 papers did not retrieve the results. Finally, 7 studies (Dai et al [14], Wu et al [15], Bai et al [20], Dai et al [21], Pan et al [22], Shi et al [23], and Ren et al [24]) were included in our meta-analysis. An additional 11 studies (Chen et al [25], Chen [26], Lin et al [27], Liu [28], Ma [29], Tu et al [30], Wen and Cui [31], Wu [32], Yang [33], Rui et al [34], and Zhou et al [35]) from the Chinese database Wanfang were included in the study. A total of 12,981 patients were included in this analysis.

### Trial Characteristics

**Figure 2.**
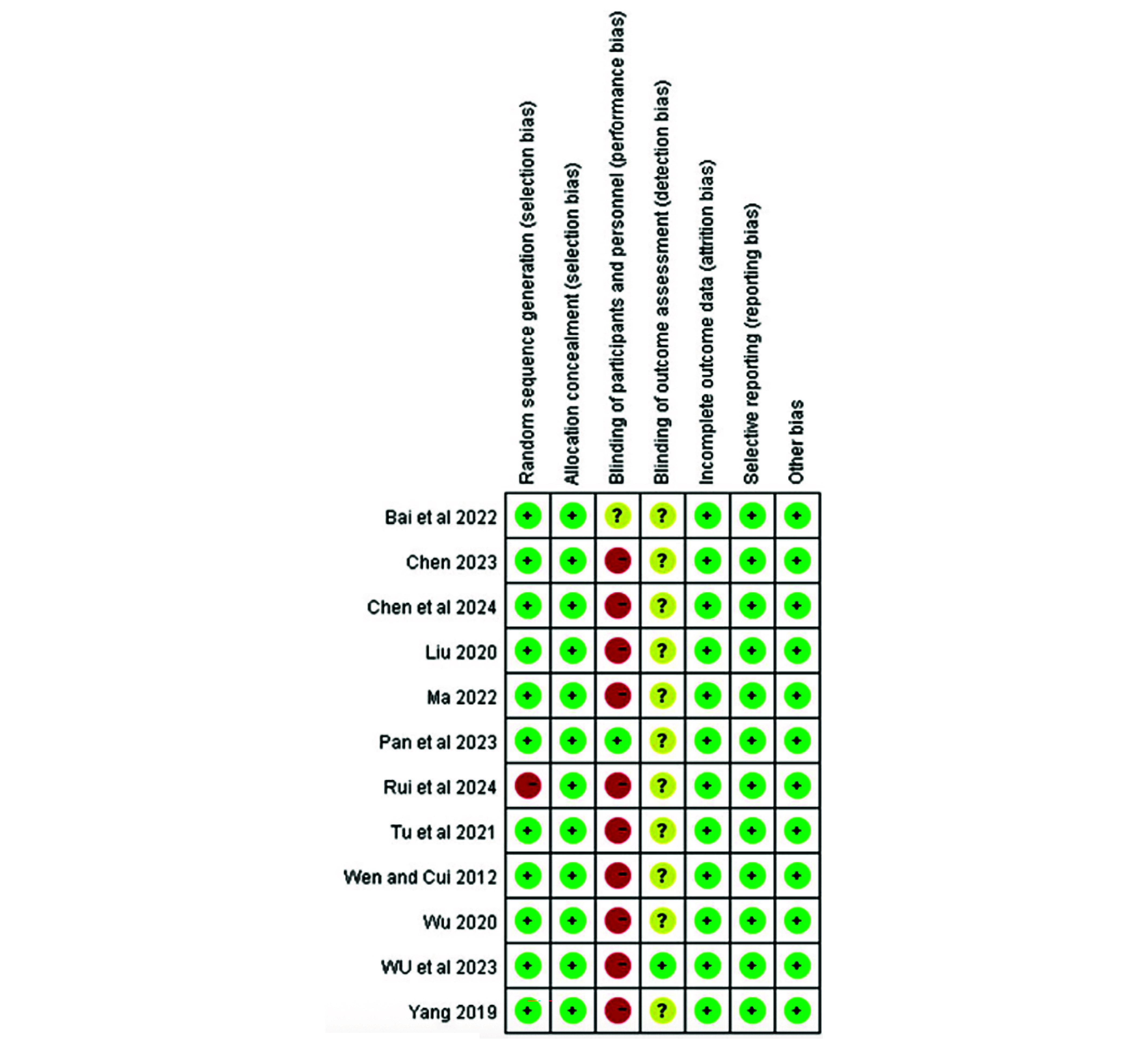
Risk of bias summary: review authors’ judgments about each risk of bias item for RCTs [15,20,22,25,26,28-34].

The mean age of the participants varied from 47.20 to 72 years. The sample sizes of the eligible studies ranged from 19 to 2723 participants. All the studies were performed in China. The literature we have included has focused primarily on populations with ischemic stroke caused by cerebral vascular atherosclerosis and CHD, which includes stable angina pectoris, acute coronary syndrome, and coronary artery stent implantation or coronary artery bypass grafting due to CHD. The dose of indobufen given to these participants ranged from 100 mg daily to 100 mg twice a day, and the daily dose of aspirin was 100 mg. The characteristics of the included studies are summarized in Table 1. Figure 2 and Table 2 provide information regarding the quality assessment for the risk of bias for each study included in the meta-analysis.

**Table 1.** Characteristics of included studies^a^.

Study	Follow-up	Population	Group	Sample size, n	Age (years), mean (SD)	Male, %	Drug and dose	Type
Bai et al [20]	1 year	CABG^b^	Intervention group	76	60.3 (6.6)	75.00	Indobufen 100 mg/day+ Clopidogrel 75 mg/day	RCT^c^
			Control group	76	59.7 (7.2)	81.60	Aspirin 100 mg/day+ Clopidogrel 75 mg/day	
Chen et al [25]	1 year	CHD^d^	Intervention group	34	66.34 (1.48)	64.70	Indobufen 100 mg twice a day + Clopidogrel 75 mg/day	RCT
			Control group	37	66.39 (1.47)	64.90	Aspirin 100 mg/day+ Clopidogrel 75 mg/day	
Chen [26]	6 months	CHD	Intervention group	42	48.10 (12.70)	38.10	Indobufen 100 mg twice a day+ Clopidogrel 75 mg/day	RCT
			Control group	42	47.20 (15.10)	40.48	Aspirin 100 mg/day+ Clopidogrel 75 mg/day	
Dai et al [14]	1 year	PCI^e^	Intervention group	662	66 (9.4)	72.20	Indobufen 100 mg twice a day + P2Y12 receptor antagonist	Cohort study
			Control group	662	66 (10.5)	70.80	Aspirin 100 mg/day+ P2Y12 receptor antagonist	
Dai et al [21]	1 year	PCI (AMI)^f^	Intervention group	224	72 (11)	62.90	Indobufen 100 mg twice a day + Clopidogrel 75 mg/day	Cohort study
			Control group	224	72 (10)	67.00	Aspirin 100 mg/day+ Clopidogrel 75 mg/day	
Lin et al [27]	1 year	CCS^g^	Intervention group	37	71.56 (2.19)	54.10	Indobufen 200 mg/day+ Clopidogrel 75 mg/day	Cohort study
			Control group	37	71.66 (2.45)	59.50	Aspirin 100 mg/day+ Clopidogrel 75 mg/day	
Liu [28]	1 year	PCI	Intervention group	30	58.5 (53.5‐66.5)	80.00	Indobufen 100 mg twice a day+ Clopidogrel 75 mg/day	RCT
			Control group	30	60 (56‐64.25)	73.30	Aspirin 100 mg/day+ Clopidogrel 75 mg/day	
Ma [29]	6 months	PCI (unstable angina)	Intervention group	41	71.76 (5.85)	68.30	Indobufen 100 mg twice a day+ Clopidogrel 75 mg/day	RCT
			Control group	41	70.51 (7.55)	65.90	Aspirin 100 mg/day+ Clopidogrel 75 mg/day	
Pan et al [22]	1 year	Stroke	Intervention group	2715	64.7 (56.3‐70.9)	64.50	Indobufen 100 mg twice a day+ Placebo	RCT
			Control group	2723	63.8 (56.0‐70.3)	64.90	Aspirin 100 mg/day+ Placebo	
Rui et al [34]	1 year	ACS^h^	Intervention group	78	68.42 (4.29)	50.00	Indobufen + Clopidogrel	Control study
			Control group	78	68.73 (4.75)	51.30	Aspirin + Clopidogrel	
Shi et al [23]	1 year	PCI (CCS)	Intervention group	52	63.07 (6.46)	96.43	Indobufen 100 mg twice a day	Crossover study
			Control group	52	63.07 (6.46)	96.43	Aspirin 100 mg/day	
Tu et al [30]	6 months	STEMI^i^	Intervention group	53	65.3 (9.8)	56.60	Indobufen 100 mg twice a day+ Ticagrelor 90 mg twice a day	RCT
			Control group	53	64.8 (10.2)	58.49	Aspirin 100 mg/day+ Ticagrelor 90 mg twice a day	
Wen and Cui [31]	6 months	PCI	Intervention group	32	58 (7)	81.25	Indobufen 100 mg twice a day+ Clopidogrel 75 mg/day	RCT
			Control group	32	57 (9)	75	Aspirin 100 mg/day+ Clopidogrel 75 mg/day	
WU [32]	1 year	PCI	Intervention group	2258	61.0 (8.3)	67.40	Indobufen 100 mg twice a day+ Clopidogrel 75 mg/day	RCT
			Control group	2293	61.2 (8.4)	63.10	Aspirin 100 mg/day+ Clopidogrel 75 mg/day	
Wu et al [15]	6 months	ACS	Intervention group	34	69.88 (4.31)	47.06	Indobufen 100 mg twice a day+ Clopidogrel 75 mg/day	RCT
			Control group	34	69.68 (4.26)	47.06	Aspirin 100 mg/day+ Clopidogrel 75 mg/day	
Yang [33]	1 year	ACS	Intervention group	38	70.34 (3.44)	65.70	Indobufen 100 mg twice a day+ Clopidogrel 75 mg/day	RCT
			Control group	38	71.03 (2.92)	65.70	Aspirin 100 mg/day+ Clopidogrel 75 mg/day	
Ren et al [24]	18 months	CABG	Intervention group	19	63.84 (7.99)	78.95	Indobufen 100 mg twice a day+ Clopidogrel 75 mg/day	Retrospective cohort study
			Control group	20	64.60 (6.72)	80.00	Aspirin 100 mg/day+ Clopidogrel 75 mg/day	
Zhou et al [35]	1 year	CABG	Intervention group	34	57.63 (2.31)	52.94	Indobufen 0.2 g/day + Clopidogrel 75 mg/day	Retrospective cohort study
			Control group	50	57.54 (2.28)	52	Aspirin 100 mg/day + Clopidogrel 75 mg/day	

a Data are presented as means (SDs) and median (IQR).

b CABG: coronary artery bypass grafting.

c RCT: randomized controlled trial.

d CHD: coronary heart disease.

e PCI: percutaneous coronary intervention.

f AMI: acute myocardial infarction.

g CCS: chronic coronary syndromes.

h ACS: acute coronary syndromes.

i STEMI: ST-segment elevation myocardial infarction.

**Table 2.** Quality assessment of non–randomized controlled trial according to Newcastle‒Ottawa Scale.

Study	Selection				Comparability		Results			Total score
	S1	S2	S3	S4	C1	C2	R1	R2	R3	
Dai et al [14]	P**^a^**	P	P	P	P	N^b^	P	P	P	8
Dai et al [21]	P	P	P	P	P	N	P	P	P	8
Lin et al [27]	P	P	P	P	P	N	P	P	N	7
Shi et al [23]	P	P	P	P	P	N	P	P	N	7
Ren et al [24]	P	P	P	P	P	N	P	P	P	8
Zhou et al [35]	P	P	P	P	P	N	P	P	N	7

a P: 1 score.

b N: zero score.

### Meta-Analysis Results

#### Major Adverse Cardiovascular and Cerebrovascular Events

Five studies reported MACCEs (Dai et al [14], Wu et al [15], Bai et al [20], Dai et al [21], and Pan et al [22]). The pooled results revealed no significant difference in MACCEs with indobufen compared with aspirin (RR 1.12, 95% CI 0.98‐1.28; *I*^2^=0%; *P*=.09; Figure S1 in Multimedia Appendix 1 [14,15,20-22]).

#### Any Bleeding

Seventeen studies reported any bleeding (Dai et al [14], Wu et al [15], Bai et al [20], Dai et al [21], Pan et al [22], Shi et al [23], Ren et al [24], Chen et al [25], Chen [26], Lin et al [27], Liu [28], Ma [29], Tu et al [30], Wen and Cui [31], Yang [33], Rui et al [34], and Zhou et al [35]). However, 1 study was excluded because there were no outcome events in either the experimental group or the control group. The combined results revealed a significant reduction in any bleeding with indobufen compared with aspirin (RR 0.54, 95% CI 0.41‐0.71; *I*^2^=51%; *P*<.0001; Figure S2 in Multimedia Appendix 1 [14,15,20-31,33-35]).

#### BARC 2/3/5 Bleeding

Five studies reported BARC type 2, 3, or 5 bleeding (BARC 2/3/5 bleeding; Dai et al [14], Wu et al [15], Dai et al [21], Shi et al [23], and Liu [28]). Two studies were excluded from the pooled meta-analysis because both groups in these studies reported zero events. The heterogeneity (*I*^2^=84%; *P*=.002) was high, so we used a random-effects model to assess the outcome. The combined results revealed a reduction in BARC 2/3/5 bleeding with indobufen compared with aspirin (RR 0.50, 95% CI 0.26‐0.94; *I*^2^=84%; *P*=.03; Figure S3 in Multimedia Appendix 1 [14,15,21,23,28]).

#### Adverse Cardiovascular Events

Thirteen studies reported adverse cardiovascular events (Bai et al [20], Ren et al [24], Chen et al [25], Chen [26], Lin et al [27], Liu [28], Ma [29], Tu et al [30], Wen and Cui [31], Wu [32], Yang [33], Rui et al [34], and Zhou et al [35]). The pooled results revealed a significant decrease in adverse cardiovascular events with indobufen compared with aspirin (RR 0.43, 95% CI 0.30‐0.61; *I*^2^=0%; *P*<.00001; Figure S4 in Multimedia Appendix 1 [20,24-35]).

#### Stroke

Seventeen studies reported stroke (Dai et al [14], Wu et al [15], Bai et al [20], Dai et al [21], Pan et al [22], Ren et al [24], Chen et al [25], Chen [26], Lin et al [27], Liu [28], Ma [29], Tu et al [30], Wen and Cui [31], Wu [32], Yang [33], Rui et al [34], and Zhou et al [35]). Five studies were excluded because of zero events. The pooled results revealed no significant difference in the incidence of stroke between patients treated with indobufen and those treated with aspirin (RR 1.07, 95% CI 0.92‐1.25; *I*^2^=0%; *P*=.37; Figure S5 in Multimedia Appendix 1 [14,15,20-22,24-35]).

#### Myocardial Infarction

Seventeen studies reported MI (Dai et al [14], Wu et al [15], Bai et al [20], Dai et al [21], Pan et al [22], Ren et al [24], Chen et al [25], Chen [26], Lin et al [27], Liu [28], Ma [29], Tu et al [30], Wen and Cui [31], Wu [32], Yang [33], Rui et al [34], and Zhou et al [35]). Four studies were excluded because of zero events. The pooled results revealed a significant decrease in MI with indobufen compared with aspirin (RR 0.60, 95% CI 0.41‐0.89; *I*^2^=0%; *P*=.01; Figure S6 in Multimedia Appendix 1 [14,15,20-22,24-35]).

#### Cardiovascular Death

Sixteen studies reported cardiovascular death (Dai et al [14], Wu et al [15], Bai et al [20], Dai et al [21], Pan et al [22], Chen et al [25], Chen [26], Lin et al [27], Liu [28], Ma [29], Tu et al [30], Wen and Cui [31], Wu [32], Yang [33], Rui et al [34], and Zhou et al [35]). Eight studies were excluded because of zero events. The pooled results revealed no significant difference in cardiovascular death between indobufen and aspirin (RR 1.13, 95% CI 0.71‐1.79; *I*^2^=0%; *P*=.61; Figure S7 in Multimedia Appendix 1 [14,15,20-22,25-35]).

### Sensitivity Analysis

**Figure 3.**
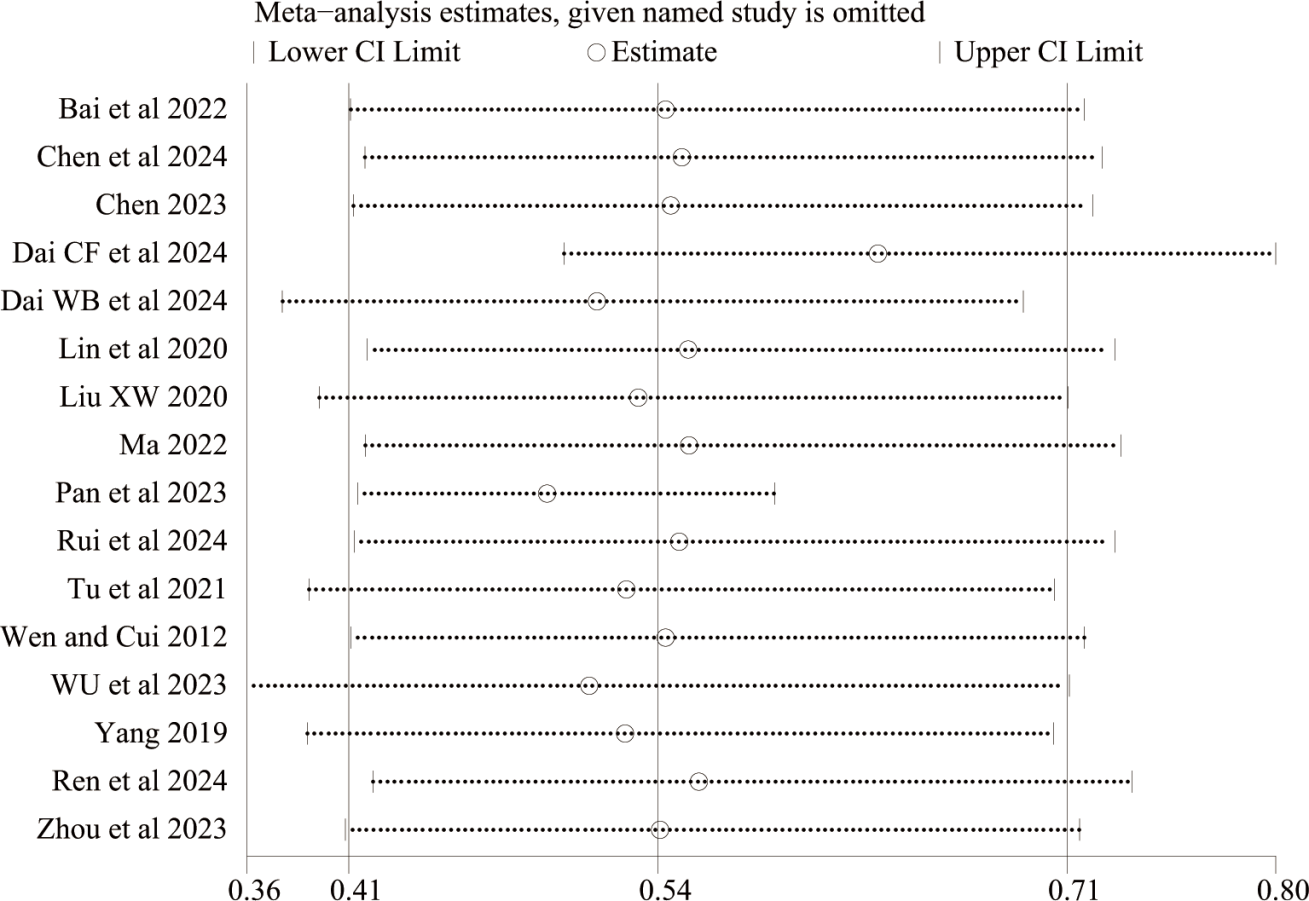
Sensitivity analysis of any bleeding [14,15,20-22,24-31,33-35].

In this meta-analysis, the results of any bleeding and BARC studies revealed a high level of heterogeneity, with *I*^2^ values of 51% and 84%, indicating substantial heterogeneity, possibly due to the small sample size. To further explore the source of this heterogeneity, we conducted a sensitivity analysis. This involved sequentially excluding individual studies to assess their influence on the overall pooled estimate. Although the limited number of studies restricts the depth of the analysis, the results showed that the pooled effect estimate remained stable, indicating relative robustness (Figures3  4). However, variations in heterogeneity were observed when individual studies were excluded, suggesting that 1 or more studies might have contributed to the observed heterogeneity. We attribute the source of the heterogeneity primarily to the use of the second antiplatelet agent in the dual antiplatelet therapy (DAPT) regimen, as well as to variations in sample size and concomitant use of proton pump inhibitors.

**Figure 4.**
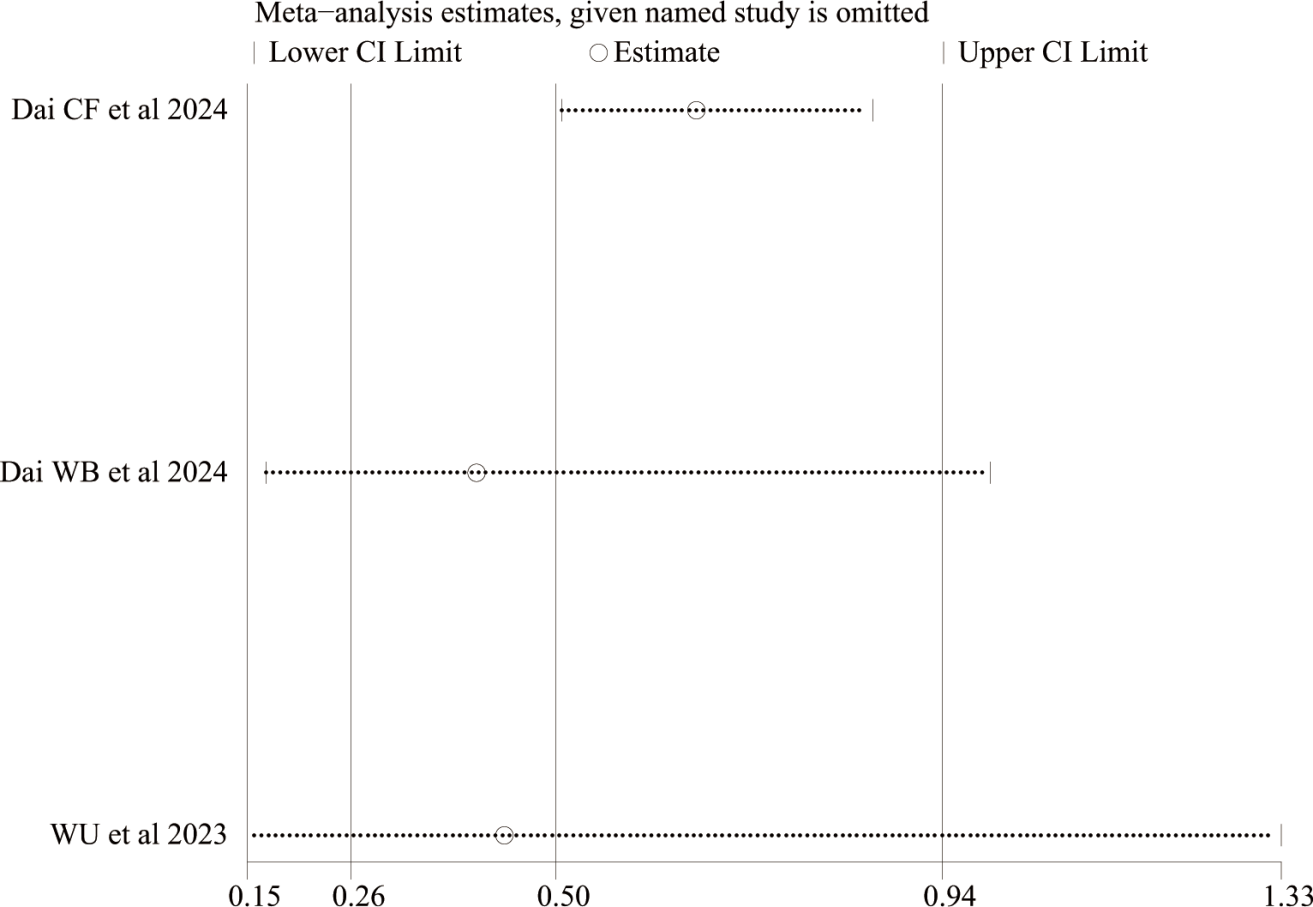
Sensitivity analysis of BARC 2/3/5 bleeding [14,15,21].

### Publication Bias

For outcomes with more than 10 papers included in the literature, we plotted funnel plots and performed Egger tests. There are 16, 13, 12, and 13 studies on outcomes of any bleeding events, adverse cardiovascular events, stroke events, and MI, respectively. The *P* values of the Egger tests for any bleeding or adverse cardiovascular events were .249 and .235, respectively, both of which are greater than .05. We believe that the publication bias is small, but the *P* values of the Egger tests for stroke and MI were .000 and .001, indicating a certain degree of publication bias. All funnel plots are included in Multimedia Appendix 1.

## Discussion

### Principal Findings

In this systematic review and meta-analysis involving 18 clinical trials and 12,981 patients, we compared antiplatelet therapy involving indobufen with that involving aspirin. Although we did not impose any language restrictions for the included studies, all the clinical trials ultimately retrieved were conducted in China. This may be because indobufen is not widely approved as a routine antiplatelet agent in Europe and the Americas and is used only as a reference drug in specific studies [36]. These results suggest that, compared with aspirin, indobufen can effectively reduce the incidence of mucosal bleeding (including both major and minor bleeding) in patients with cardiovascular and cerebrovascular atherosclerosis. Additionally, on the basis of the studies included in our analysis, indobufen was shown to reduce the incidence of adverse cardiovascular events (a composite outcome of coronary thrombus reformation, heart failure, MI, stroke, angina pectoris, and cardiovascular death) as well as MI. However, MACCEs, stroke, and cardiovascular death rates were similar between the 2 groups, which may be related to the limited follow-up time. The heterogeneity for any bleeding and BARC 2/3/5 bleeding was high, but the sensitivity analysis revealed no difference in outcomes. There was significant publication bias for stroke, MI, and cardiovascular death. The effects of indobufen on MACCEs, stroke, and cardiovascular death were similar to those of aspirin, demonstrating no inferiority in efficacy.

Antiplatelet therapy in patients with atherosclerosis remains a topic of considerable debate. Current guidelines recommend aspirin for secondary prevention in patients with coronary atherosclerotic heart disease, and DAPT is advised for patients with a high risk of thrombosis and a low risk of bleeding or those undergoing interventional treatment [37]. For patients who require DAPT but are intolerant to aspirin, indobufen is being investigated as a potential alternative to aspirin [38,39].

The antiplatelet effect of aspirin is achieved by irreversibly inhibiting and permanently blocking COX-1 in platelets, thereby preventing the formation of thromboxane A2 (TXA2) [40]. This effect lasts for the lifespan of the platelet (7‐10 days) [41]. In contrast, indobufen is a reversible COX-1 inhibitor, meaning that it temporarily inhibits the enzyme but does not cause persistent inhibition [42]. This reversible action allows for shorter-term effects on platelet function than does aspirin [43]. The inhibitory effects of indobufen on platelet function returned to baseline within 24 hours after discontinuation. Additionally, indobufen does not substantially impact prostacyclin, potentially minimizing the risk of aspirin-gastric side effects [25]. While both drugs seek to reduce platelet aggregation and associated thrombosis, the irreversible inhibition of aspirin leads to protracted platelet inhibition, whereas indobufen offers a reversible and potentially safer alternative, particularly in patients at greater risk for bleeding.

Aspirin’s first-line status in the prevention and treatment of cardiovascular and cerebrovascular diseases is supported by a large body of evidence-based research [10,44,45]. In comparison, although the clinical application of indobufen has evolved, it remains very limited at present. Initially, owing to its reversible inhibition of COX, it was not recommended, as it was considered insufficient to effectively inhibit platelet aggregation in vivo [46]. However, subsequent clinical trials have led some meta-analyses to consider indobufen similarly to aspirin. Cataldo G et al [47], in their combined analysis of 2 multicenter coronary artery bypass grafting studies, concluded that indobufen is as effective as aspirin and dipyridamole in preventing graft occlusion. Bhana and McClellan [48], in their review, suggested that indobufen may be an effective alternative to aspirin for patients with nonrheumatic atrial fibrillation who have contraindications to anticoagulation or a greater risk of bleeding. In a meta-analysis of 9 clinical studies, Zhang et al [49] reported that indobufen had fewer gastrointestinal reactions, a lower risk of bleeding, and could be a viable option for patients at high risk of bleeding or with gastrointestinal ulcers.

### Strengths and Limitations

The strengths of our study include the following: compared with previous studies, our meta-analysis incorporated a larger number of clinical trials and patients with homogeneous outcome definitions and a large patient population, making the results more generalizable to the broader population. Additionally, the follow-up duration in all the included studies exceeded 6 months, enhancing the credibility of our findings. We also conducted publication bias and sensitivity analyses to further emphasize the reliability of this meta-analysis.

There are several limitations to this study. All the studies included focused on Chinese populations, which may limit the generalizability of our findings to other regions of the world. But we believe that with the accumulation of further clinical evidence in China, the safety profile of indobufen as a potential alternative to aspirin will gain stronger support, paving the way for the development of larger multicenter studies with longer follow-up periods and more diverse populations across different regions—including global clinical trials. Additionally, because antiplatelet therapy is a long-term treatment requiring good patient adherence, the included trials were open-label, which might raise concerns about potential bias, although none of the studies carried a high risk of bias. Owing to the lack of available data, we were unable to perform subgroup analyses on the basis of individual patient characteristics, and the efficacy and safety of indobufen in specific populations require further investigation.

### Conclusions

Our meta-analysis indicates that, compared with aspirin, indobufen offers a safer profile for antiplatelet therapy in patients with cardiovascular and cerebrovascular atherosclerosis, as it reduces the incidence of mucosal bleeding. In terms of efficacy, indobufen is associated with a lower incidence of adverse cardiovascular events and MI, while its impact on MACCEs and cardiovascular mortality is similar to that of aspirin. Indobufen demonstrated no overall inferiority in effectiveness, with superior results in some aspects (adverse cardiovascular events and MI). This provides additional robust evidence supporting the feasibility of using indobufen as an alternative antiplatelet therapy, particularly in patients who are intolerant to aspirin.

## Supplementary material

10.2196/75363 Multimedia Appendix 1 Forest plots for the outcomes. Figure S1: Major adverse cardiovascular and cerebrovascular events (risk ratio 1.12, CI 0.98-1.28; *P*=.09); Figure S2: any bleeding events (risk ratio 0.54, CI 0.41-0.71; *P*<.0001); Figure S3: Bleeding Academic Research Consortium 2/3/5/5 bleeding (risk ratio 0.50, CI 0.26-0.94; *P*=.03); Figure S4: adverse cardiovascular events (risk ratio 0.43, CI 0.30-0.61; *P*<.00001); Figure S5: stroke events (risk ratio 1.07, CI 0.92-1.25; *P*=.37); Figure S6: myocardial infarction events (risk ratio 0.60, CI 0.41-0.89; *P*=.01); and Figure S7: cardiovascular death associated with the use of indobufen and aspirin (risk ratio 1.13, CI 0.71-1.79; *P*=.61). RR: risk ratio.

10.2196/75363 Checklist 1 PRISMA (Preferred Reporting Items for Systematic Reviews and Meta-Analyses) checklist.

## Abbreviations

BARC: Bleeding Academic Research Consortium
CHD: coronary heart disease
COX-1: cyclooxygenase-1
DAPT: dual antiplatelet therapy
MACCE: major adverse cardiovascular and cerebrovascular event
MeSH: Medical Subject Headings
MI: myocardial infarction
PRISMA: Preferred Reporting Items for Systematic Reviews and Meta-Analyses
RCT: randomized controlled trial
RR: risk ratio
TXA2: thromboxane A2
