# Supplementary material for: Safety and Efficacy of Aspirin and Indobufen in the Treatment of Atherosclerotic Diseases: Systematic Review and Meta-Analysis
Source: Interact J Med Res. 2025 Aug 20;14:e75363. doi: 10.2196/75363 (PMC12391900; doi:10.2196/75363)
Supplement: Multimedia Appendix 1 [file ijmr-v14-e75363-s001.pdf]

S1

Study or Subgroup	Experimental Events	Experimental Total	Control Events	Control Total	Weight	Risk Ratio M-H, Fixed, 95% CI
Bai et al 2022	1	76	2	76	0.5%	0.50 [0.05, 5.40]
Dai CF et al 2024	43	662	43	662	11.7%	1.00 [0.66, 1.50]
Dai WB et al 2024	45	224	33	224	9.0%	1.36 [0.91, 2.05]
Pan et al 2023	287	2715	257	2723	70.0%	1.12 [0.95, 1.31]
WU et al 2023	34	2258	32	2293	8.7%	1.08 [0.67, 1.74]
**Total (95% CI)**		**5935**		**5978**	**100.0%**	**1.12 [0.98, 1.28]**
Total events	410		367			

Heterogeneity: Chi² = 1.65, df = 4 (P = 0.80); I² = 0%
Test for overall effect: Z = 1.67 (P = 0.09)

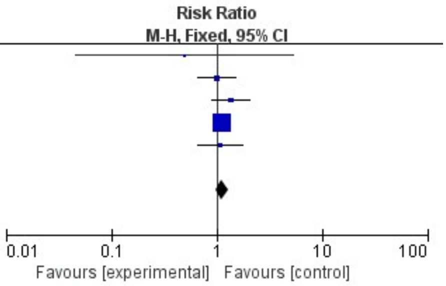

S2

Study or Subgroup	Experimental Events	Experimental Total	Control Events	Control Total	Weight	Risk Ratio M-H, Random, 95% CI
Bai et al 2022	0	76	2	76	0.8%	0.20 [0.01, 4.10]
Chen 2023	1	42	4	42	1.6%	0.25 [0.03, 2.14]
Chen et al 2024	1	34	6	37	1.7%	0.18 [0.02, 1.43]
Dai CF et al 2024	72	662	185	662	16.6%	0.39 [0.30, 0.50]
Dai WB et al 2024	18	224	23	224	10.4%	0.78 [0.43, 1.41]
Lin et al 2020	2	37	8	37	3.0%	0.25 [0.06, 1.10]
Liu 2020	5	30	8	30	5.6%	0.63 [0.23, 1.69]
Ma 2022	3	41	10	41	4.2%	0.30 [0.09, 1.01]
Pan et al 2023	81	2715	83	2723	15.6%	0.98 [0.72, 1.32]
Ren et al 2024	3	19	11	20	4.8%	0.29 [0.09, 0.87]
Rui et al 2024	4	78	11	78	4.9%	0.36 [0.12, 1.09]
Shi et al 2022	0	52	0	52		Not estimable
Tu et al 2021	7	53	10	53	6.6%	0.70 [0.29, 1.70]
Wen and Cui 2012	0	32	2	32	0.8%	0.20 [0.01, 4.01]
WU et al 2023	67	2258	108	2293	15.7%	0.63 [0.47, 0.85]
Yang 2019	7	38	10	38	6.9%	0.70 [0.30, 1.65]
Zhou et al 2023	0	34	2	50	0.8%	0.29 [0.01, 5.89]
**Total (95% CI)**		**6425**		**6488**	**100.0%**	**0.54 [0.41, 0.71]**
Total events	271		483			

Heterogeneity: Tau² = 0.11; Chi² = 30.55, df = 15 (P = 0.01); I² = 51%
Test for overall effect: Z = 4.33 (P < 0.0001)

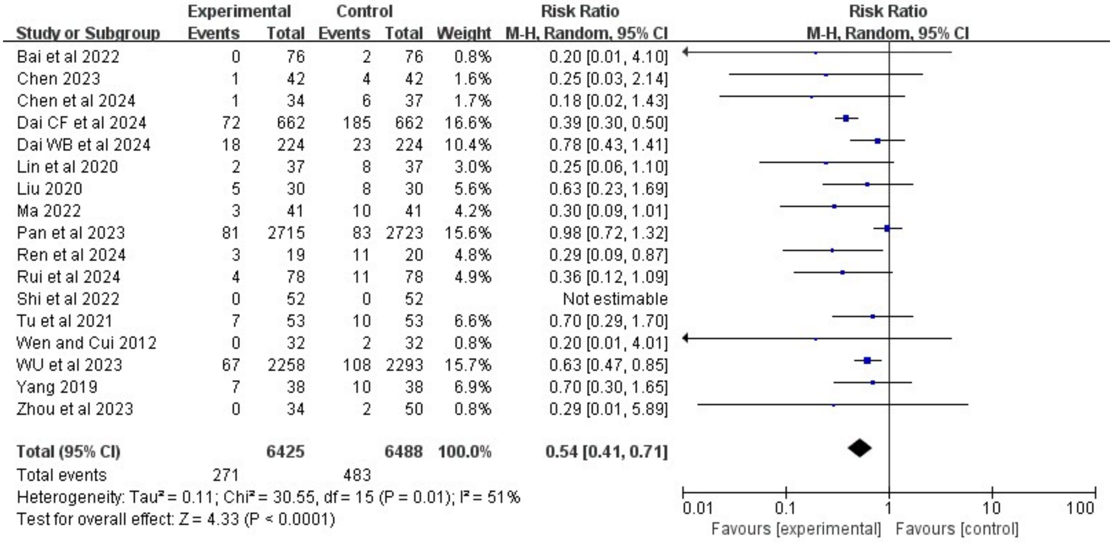

S3

Study or Subgroup	Experimental Events	Experimental Total	Control Events	Control Total	Weight	Risk Ratio M-H, Random, 95% CI
Dai CF et al 2024	20	662	79	662	32.9%	0.25 [0.16, 0.41]
Dai WB et al 2024	18	224	23	224	30.0%	0.78 [0.43, 1.41]
Liu 2020	0	0	0	0		Not estimable
Shi et al 2022	0	52	0	52		Not estimable
WU et al 2023	67	2258	108	2293	37.1%	0.63 [0.47, 0.85]
**Total (95% CI)**		**3196**		**3231**	**100.0%**	**0.50 [0.26, 0.94]**
Total events	105		210			

Heterogeneity: Tau² = 0.26; Chi² = 12.23, df = 2 (P = 0.002); I² = 84%
Test for overall effect: Z = 2.14 (P = 0.03)

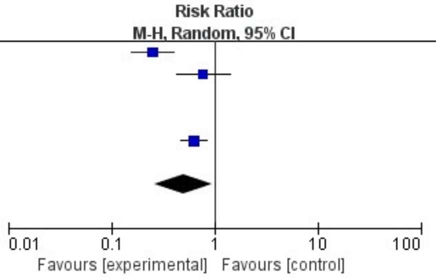

S4

Study or Subgroup	Experimental Events	Experimental Total	Control Events	Control Total	Weight	Risk Ratio M-H, Fixed, 95% CI
Bai et al 2022	4	76	4	76	4.4%	1.00 [0.26, 3.85]
Chen 2023	7	42	17	42	18.8%	0.41 [0.19, 0.89]
Chen et al 2024	0	34	4	37	4.8%	0.12 [0.01, 2.16]
Lin et al 2020	1	37	2	37	2.2%	0.50 [0.05, 5.28]
Liu 2020	0	30	1	30	1.7%	0.33 [0.01, 7.87]
Ma 2022	1	41	6	41	6.6%	0.17 [0.02, 1.32]
Ren et al 2024	0	19	5	20	5.9%	0.10 [0.01, 1.62]
Rui et al 2024	5	78	11	78	12.1%	0.45 [0.17, 1.25]
Tu et al 2021	6	53	14	53	15.5%	0.43 [0.18, 1.03]
Wen and Cui 2012	0	32	1	32	1.7%	0.33 [0.01, 7.89]
Wu 2020	6	34	14	34	15.5%	0.43 [0.19, 0.98]
Yang 2019	3	38	5	38	5.5%	0.60 [0.15, 2.34]
Zhou et al 2023	3	34	6	50	5.4%	0.74 [0.20, 2.74]
**Total (95% CI)**		**548**		**568**	**100.0%**	**0.43 [0.30, 0.61]**
Total events	36		90			

Heterogeneity: Chi² = 5.12, df = 12 (P = 0.95); I² = 0%
Test for overall effect: Z = 4.77 (P < 0.00001)

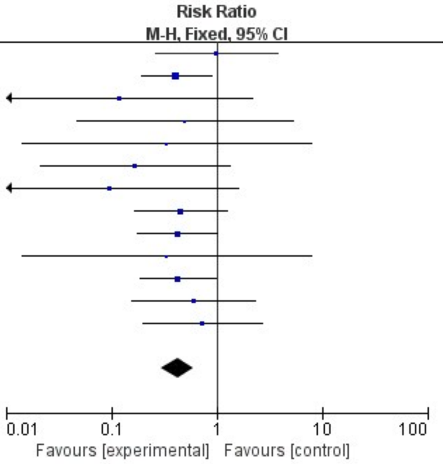

S5

Study or Subgroup	Experimental Events	Experimental Total	Control Events	Control Total	Weight	Risk Ratio M-H, Fixed, 95% CI
Bai et al 2022	0	76	0	76		Not estimable
Chen 2023	1	42	3	42	1.0%	0.33 [0.04, 3.08]
Chen et al 2024	0	34	4	37	1.5%	0.12 [0.01, 2.16]
Dai CF et al 2024	3	662	4	662	1.4%	0.75 [0.17, 3.34]
Dai WB et al 2024	8	224	8	224	2.7%	1.00 [0.38, 2.62]
Lin et al 2020	0	37	0	37		Not estimable
Liu 2020	0	30	0	30		Not estimable
Ma 2022	0	41	2	41	0.9%	0.20 [0.01, 4.04]
Pan et al 2023	279	2715	242	2723	82.4%	1.16 [0.98, 1.36]
Ren et al 2024	0	19	1	20	0.5%	0.35 [0.02, 8.10]
Rui et al 2024	1	78	3	78	1.0%	0.33 [0.04, 3.14]
Tu et al 2021	1	53	2	53	0.7%	0.50 [0.05, 5.35]
Wen and Cui 2012	0	32	0	32		Not estimable
Wu 2020	1	34	3	34	1.0%	0.33 [0.04, 3.05]
WU et al 2023	18	2258	19	2293	6.4%	0.96 [0.51, 1.83]
Yang 2019	0	38	1	38	0.5%	0.33 [0.01, 7.93]
Zhou et al 2023	0	34	0	50		Not estimable
**Total (95% CI)**		**6407**		**6470**	**100.0%**	**1.07 [0.92, 1.25]**
Total events	312		292			

Heterogeneity: Chi² = 9.15, df = 11 (P = 0.61); I² = 0%
Test for overall effect: Z = 0.90 (P = 0.37)

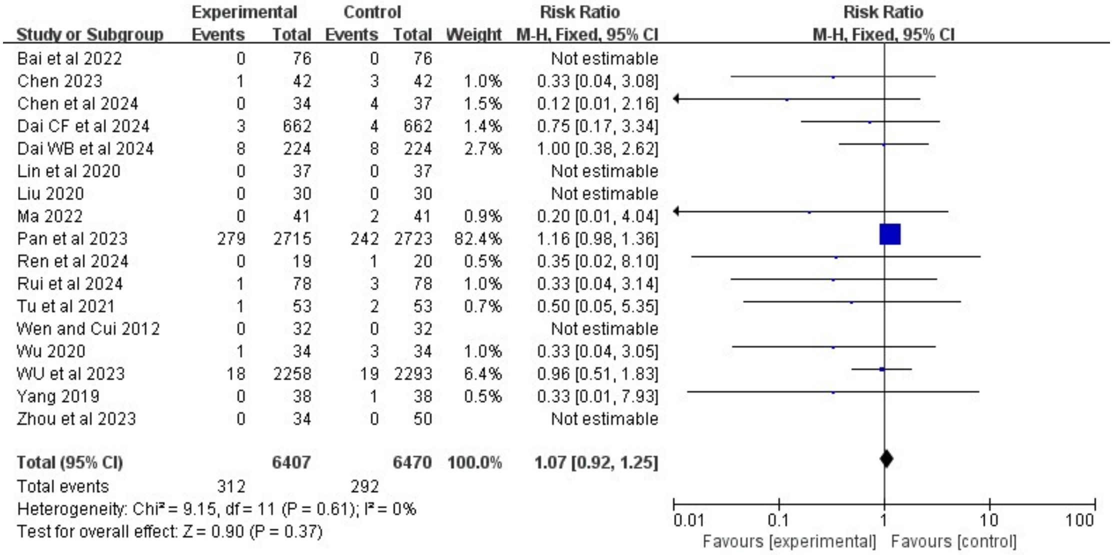

S6

Study or Subgroup	Experimental Events	Experimental Total	Control Events	Control Total	Weight	Risk Ratio M-H, Fixed, 95% CI
Bai et al 2022	0	76	1	76	2.3%	0.33 [0.01, 8.06]
Chen 2023	2	42	4	42	6.2%	0.50 [0.10, 2.58]
Chen et al 2024	0	34	0	37		Not estimable
Dai CF et al 2024	3	662	7	662	10.8%	0.43 [0.11, 1.65]
Dai WB et al 2024	8	224	9	224	13.9%	0.89 [0.35, 2.26]
Lin et al 2020	1	37	2	37	3.1%	0.50 [0.05, 5.28]
Liu 2020	0	30	0	30		Not estimable
Ma 2022	1	41	4	41	6.2%	0.25 [0.03, 2.14]
Pan et al 2023	5	2715	9	2723	13.9%	0.56 [0.19, 1.66]
Ren et al 2024	0	19	2	20	3.8%	0.21 [0.01, 4.11]
Rui et al 2024	3	78	5	78	7.7%	0.60 [0.15, 2.42]
Tu et al 2021	3	53	5	53	7.7%	0.60 [0.15, 2.38]
Wen and Cui 2012	0	32	0	32		Not estimable
Wu 2020	1	34	3	34	4.6%	0.33 [0.04, 3.05]
WU et al 2023	9	2258	10	2293	15.3%	0.91 [0.37, 2.24]
Yang 2019	2	38	3	38	4.6%	0.67 [0.12, 3.77]
Zhou et al 2023	0	34	0	50		Not estimable
**Total (95% CI)**		**6407**		**6470**	**100.0%**	**0.60 [0.41, 0.89]**
Total events	38		64			

Heterogeneity: Chi² = 3.38, df = 12 (P = 0.99); I² = 0%
Test for overall effect: Z = 2.54 (P = 0.01)

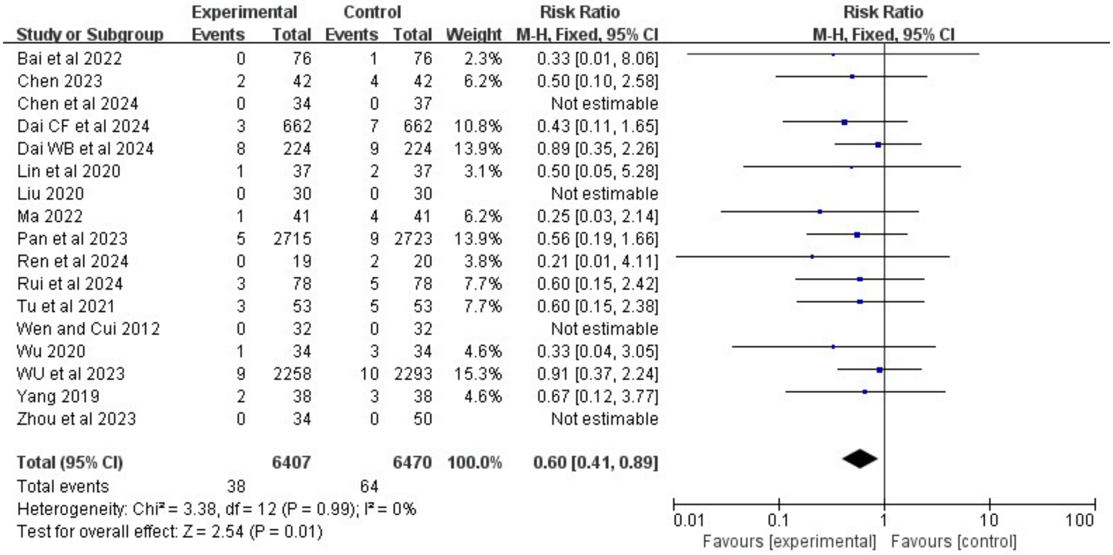

S7

Study or Subgroup	Experimental Events	Experimental Total	Control Events	Control Total	Weight	Risk Ratio M-H, Fixed, 95% CI
Bai et al 2022	1	76	1	76	3.1%	1.00 [0.06, 15.70]
Chen 2023	0	42	0	42		Not estimable
Chen et al 2024	0	34	0	37		Not estimable
Dai CF et al 2024	4	662	4	662	12.5%	1.00 [0.25, 3.98]
Dai WB et al 2024	25	224	16	224	50.0%	1.56 [0.86, 2.85]
Lin et al 2020	0	37	0	37		Not estimable
Liu 2020	0	30	1	30	4.7%	0.33 [0.01, 7.87]
Ma 2022	0	41	0	41		Not estimable
Pan et al 2023	0	2715	0	2723		Not estimable
Rui et al 2024	1	78	3	78	9.4%	0.33 [0.04, 3.14]
Tu et al 2021	0	53	0	53		Not estimable
Wen and Cui 2012	0	32	1	32	4.7%	0.33 [0.01, 7.89]
Wu 2020	0	34	0	34		Not estimable
WU et al 2023	3	2258	4	2293	12.4%	0.76 [0.17, 3.40]
Yang 2019	1	38	1	38	3.1%	1.00 [0.06, 15.41]
Zhou et al 2023	0	34	0	50		Not estimable
**Total (95% CI)**		**6388**		**6450**	**100.0%**	**1.13 [0.71, 1.79]**
Total events	35		31			

Heterogeneity: Chi² = 3.72, df = 7 (P = 0.81); I² = 0%
Test for overall effect: Z = 0.51 (P = 0.61)

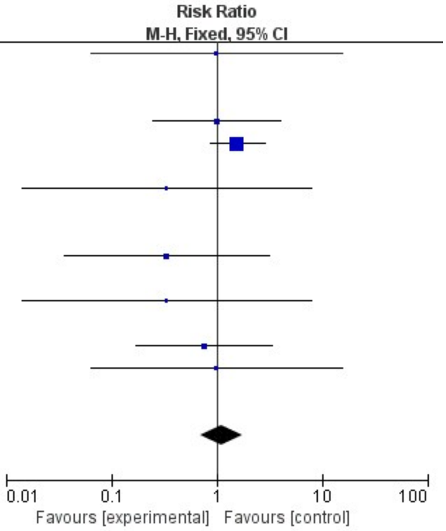

Reference:

[14]Dai C, Liu M, Yang Z, et al. Real-world performance of indobufen versus aspirin after percutaneous coronary intervention: insights from the ASPIRATION registry. BMC Med. Apr 2, 2024;22(1):148. [doi: 10.1186/s12916-024-03374-3] [Medline: 38561738]

[15]Wu H, Xu L, Zhao X, et al. Indobufen or aspirin on top of clopidogrel after coronary drug-eluting stent i Aspirin on Top of Clopidogrel After Coronary Drug-Eluting Stent Implantation (OPTION): a randomized, open-label, end point–blinded, noninferiority trialA Randomized, Open-Label, End Point–Blinded, Noninferiority Trial. Circulation. Jan 17, 2023;147(3):212-222. [doi: 10.1161/CIRCULATIONAHA.122.062762]

[20]Bai C, Li JX, Yu Y, et al. A randomized controlled trial of indobufen versus aspirin in the prevention of bridging restenosis after coronary artery bypass grafting. Zhonghua Xin Xue Guan Bing Za Zhi. May 24, 2022;50(5):466-470. [doi: 10.3760/cma.j.cn112148-20210701-00560] [Medline: 35589595]

[21]Dai WB, Ren JY, Hu ST, et al. The safety and efficacy of indobufen or aspirin combined with clopidogrel in patients with acute myocardial infarction after percutaneous coronary intervention. Platelets. Dec 2024;35(1):2364748. [doi: 10.1080/09537104.2024.2364748] [Medline: 39115322]

[22]Pan Y, Meng X, Yuan B, et al. Indobufen versus aspirin in patients with acute ischaemic stroke in China (INSURE): a randomised, double-blind, double-dummy, active control, non-inferiority trial. Lancet Neurol. Jun 2023;22(6):485-493. [doi: 10.1016/S1474-4422(23)00113-8] [Medline: 37121237]

[23]Shi QP, Luo XY, Zhang B, et al. Effect of indobufen vs. aspirin on platelet accumulation in patients with stable coronary heart disease after percutaneous coronary intervention: aAn open-label crossover study. Front Pharmacol. 2022;13:950719. [doi: 10.3389/fphar.2022.950719] [Medline: 36052139]

[24]Ren Y, Zhu Y, Yan Q, Jin H, Luo H. Multicenter retrospective cohort study demonstrates superior safety profile of indobufen over aspirin for Post-CABG antiplatelet therapy. Front Pharmacol. 2024;15:1474150. [doi: 10.3389/fphar.2024.1474150] [Medline: 39403145]

[25]Chen B, Yang YJ, Song JJ. Observation on the effect of indobufen in the treatment of coronary heart disease combined with non valvular atrial fibrillation. Technol Health. 2024;3(11):45-48.

[26]Chen YC. The effect of indobufen combined with clopidogrel on myocardial perfusiEffect of Indobufen combined with Clopidogrel on myocardial Perfusion, NT-proBNP, and MACE in patients with coronary heart disease after Patients with coronary Heart Disease after PCI. Clin Res. 2023;31(10):53-56. [doi: 10.12385/j.issn.2096-1278(2023)10-0053-04]

[27]Lin F, Huang DS, Fu MW. The effect of clopidogrel and indobufen dual antiplatelet therapy on patients with stable coronary heart disease after coronary stenting. China Prac Med. 2020;15(31):95-97. [doi: 10.14163/j.cnki.11-5547/r.2020.31.040]

[28]Liu XW. Indobufen versus aspirin combined with clopidogrel used for dual antiplatelet therapy after drug-eluting stent implantation. Inner Mongolia Medical University, 2020. (https://med.wanfangdata.com.cn/Paper/Detail?id=DegreePaper_D02338473&dbid=WF_XW).
[29]Ma K. Efficacy and safety study of indobufen combined with clopidogrel in patients with unstable angina pectoris after PCI. Hebei Medical University; 2022.
[30]Tu ZT, Pei F, Yu CQ. Effect of ticagrelor combined with indobufen on clinical efficacy and prognosis of patients with acute ST-segment elevation myocardial infarction. Chongqing Med. 2021;50(21):3660-3663. [doi:10.3969/j.issn.1671-8348.2021.21.013]
[31]Wen JL, Cui XY. To explore the prevention effect of indobufen on PCI patients with coronary artery restenosis. J TCM Univ Hunan. 2012;32(10):29-30. [doi: 10.3969/j.issn.1674-070X.2012.10.013.029.03]
[32]WU YQ. Clinical comparison of indobufen and aspirin combined with clopidogrel in the treatment of acute coronary syndromeComparison of Indobufen and Aspirin Combined with Clopidogrel in the Treatment of Acute Coronary Syndrome. Chin Foreign Med Res. 2020;18(32):40-42. [doi: 10.14033/j.cnki.cfmr.2020.32.015]
[33]Yang S. Efficacy and safety of indobufen plus clopidogrel for postoperative PCI in patients with ACS who are at high risk for gastrointestinal bleeding. Hebei Medical University; 2019.
[34]Rui SH, Wang CF, Li YH. Application of indobuprofen combined with clopidogrel in patients with ACS after PCI. Chin J Health Care Med. ;26(3):270-273. [doi: 10.3969/j.issn.1674-3245.2024.03.004]
[35]Zhou CY, Su GB, Liu XC. Clinical efficacy and safety of indobufen combined with clopidogrel in preventing vascular restenosis in saphenous vein grafts following coronary artery bypass grafting. Chin J Ration Drug Use. 2023;20(5):122-126. [doi: 10.3969/j.issn.2096-3327.2023.05.020]
